# Detection of rare single nucleotide variants affecting genes in the DNA repair pathways in hereditary breast cancer

**DOI:** 10.1186/1471-2164-15-S2-P20

**Published:** 2014-04-02

**Authors:** Shireen Hussein, Adnan Merdad, Jaudah Al-Maghrabi, Mamdooh A Gari, Fatma Al-Thubaiti, Ibtessam R Hussein, Adeel G Chaudhary, Adel M Abuzenadah, Hanaa Tashkandi, Shadi Al-Khayyat, Taha Kumosani, Mohammed H Al-Qahtani, Ashraf Dallol

**Affiliations:** 1 Center of Excellence in Genomic Medicine Research, King Abdulaziz University, Jeddah, Kingdom of Saudi Arabia; 2 Department of Surgery, King Abdulaziz University Hospital, Kingdom of Saudi Arabia; 3 Department of Pathology, King Abdulaziz University Hospital, Kingdom of Saudi Arabia; 4 Department of Oncology, King Abdulaziz University Hospital, Kingdom of Saudi Arabia; 5 Department of Biochemistry, Faculty of Science, King Abdulaziz University, Kingdom of Saudi Arabia

## Background

Patients with hereditary breast cancer constitute a considerable fraction of overall breast cancer sufferers. The contribution of genetic factors to the development of breast cancer in the admixed and highly consanguineous population of the western region of Saudi Arabia is thought to be significant as the disease is early onset [1]. The current protocols of continuous clinical follow-up of relatives of such patients are costly and cause a burden on the usually over-stretched medical resources. Discovering the significant contribution of BRCA1/2 mutations to breast cancer susceptibility allowed for the design of genetic tests that allows the medical practitioner to focus the care for those who need it most. However, BRCA1/2 mutations do not account for all breast cancer susceptibility genes and there are other genetic factors, known and unknown that may play a role in the development of such disease.

## Materials and methods

We have performed whole-exome sequencing of seven cases of breast cancer patients with positive family history of the disease using the Agilent SureSelect™ Whole-Exome Enrichment kit and sequencing on the SOLiD™ platform.

## Results

In addition to identifying two rare or novel mutations in BRCA2, we have identified several coding single nucleotide variations that affect genes controlling DNA repair in the BRCA1/2 pathway. The disruption of these pathways is very likely to contribute to breast cancer susceptibility.

## Conclusions

Our findings suggest that whole exome sequencing is a powerful tool for identifying mutations associated with hereditary breast cancer that might be missed by using other classical genetic testing strategies. Moreover, this will guide the treatment of breast cancer patients who have failed to respond to first-line therapies, thus, it is a great leap towards applying personalized medicine in Saudi Arabia.

This project is partly funded by NSTIP grant 09-BIO-818-03.
